# Simulation of semilunar valve function: computer-aided design, 3D printing and flow assessment with MR

**DOI:** 10.1186/s41205-020-0057-8

**Published:** 2020-02-03

**Authors:** Nabil Hussein, Pascal Voyer-Nguyen, Sharon Portnoy, Brandon Peel, Eric Schrauben, Christopher Macgowan, Shi-Joon Yoo

**Affiliations:** 1 Division of Cardiology, Department of Paediatrics and Division of Cardiovascular Surgery, Department of Surgery, Hospital for Sick Children, University of Toronto, Toronto, Ontario Canada; 2 Center for Image-Guided Innovation and Therapeutic Intervention (CIGITI), Hospital for Sick Children, University of Toronto, Toronto, Ontario Canada; 3 Medical Biophysics & Medical Imaging, Hospital for Sick Children, University of Toronto, Toronto, Ontario Canada; 4 Department of Diagnostic Imaging and Division of Cardiology, Department of Paediatrics Hospital for Sick Children, University of Toronto, 555 University Avenue, Toronto, Ontario M5G1X8 Canada

**Keywords:** Aortic valve, 4D-flow MRI, 3D-printing, Computer-aided design

## Abstract

**Background:**

The structure of the valve leaflets and sinuses are crucial in supporting the proper function of the semilunar valve and ensuring leaflet durability. Therefore, an enhanced understanding of the structural characteristics of the semilunar valves is fundamental to the evaluation and staging of semilunar valve pathology, as well as the development of prosthetic or bioprosthetic valves. This paper illustrates the process of combining computer-aided design (CAD), 3D printing and flow assessment with 4-dimensional flow magnetic resonance imaging (MRI) to provide detailed assessment of the structural and hemodynamic characteristics of the normal semilunar valve.

**Methods:**

Previously published geometric data on the aortic valve was used to model the ‘normal’ tricuspid aortic valve with a CAD software package and 3D printed. An MRI compatible flow pump with the capacity to mimic physiological flows was connected to the phantom. A peak flow rate of 100 mL/s and heart rate of 60 beats per minute were used. MRI measurements included cine imaging, 2D and 4D phase-contrast imaging to assess valve motion, flow velocity and complex flow patterns.

**Results:**

Cine MRI data showed normal valve function and competency throughout the cardiac cycle in the 3D-printed phantom. Quantitative analysis of 4D Flow data showed net flow through 2D planes proximal and distal to the valve were very consistent (26.03 mL/s and 26.09 mL/s, respectively). Measurements of net flow value agreed closely with the flow waveform provided to the pump (27.74 mL/s), confirming 4D flow acquisition in relation to the pump output. Peak flow values proximal and distal to the valve were 78.4 mL/s and 63.3 mL/s, respectively.

Particle traces of flow from 4D-phase contrast MRI data demonstrated flow through the valve into the ascending aorta and vortices within the aortic sinuses, which are expected during ventricular diastole.

**Conclusion:**

In this proof of concept study, we have demonstrated the ability to generate physiological 3D-printed aortic valve phantoms and evaluate their function with cine- and 4D Flow MRI. This technology can work synergistically with promising tissue engineering research to develop optimal aortic valve replacements, which closely reproduces the complex function of the normal aortic valve.

## Introduction

The aortic and pulmonary valves are collectively called the semilunar valves as they consist of three semilunar shaped leaflets that show a gentle concave curvature when viewed from above. These valves are contained within the arterial root, which has three visible round outward protrusions called the sinuses of Valsalva. The structure of both the valve leaflets and sinuses are crucial in supporting the proper function of the semilunar valve and ensuring durability of the valve leaflets, which open and close approximately 100,000 times daily without any resting period. An enhanced understanding of the structural characteristics of the semilunar valves is therefore fundamental to the evaluation and staging of semilunar valve pathology, as well as the development of prosthetic or bioprosthetic valves. We hypothesized that a combination of computer-aided design (CAD), 3D printing and flow assessment with magnetic resonance imaging (MRI) would provide an unprecedented opportunity for detailed assessment of the structural and hemodynamic characteristics of both normal semilunar valves and pathologic conditions such as isolated aortic and pulmonary valve diseases and connective tissue diseases affecting the arterial roots. This paper illustrates the process of CAD and 3D printing of a semilunar valve phantom and the assessment of valve function using 4-dimensional (4D) flow MRI.

## Method

**Fig. 1:**
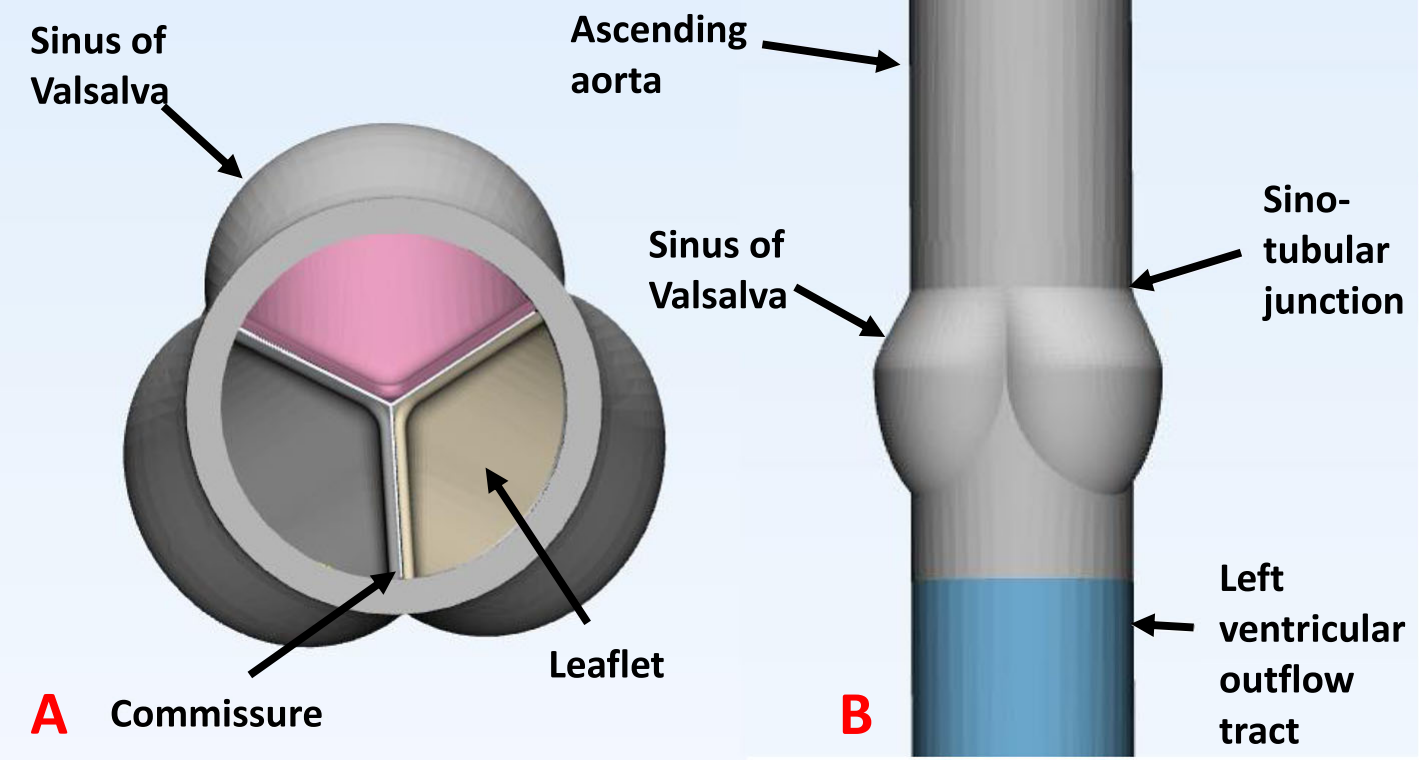
Normal tricuspid aortic valve modelled using computer aided design based on geometry obtained from literature. **a** Superior view looking down the ascending aorta to visualize the aortic valve leaflets. **b** Lateral view of aortic valve phantom incorporating aortic root structures

**Fig. 2:**
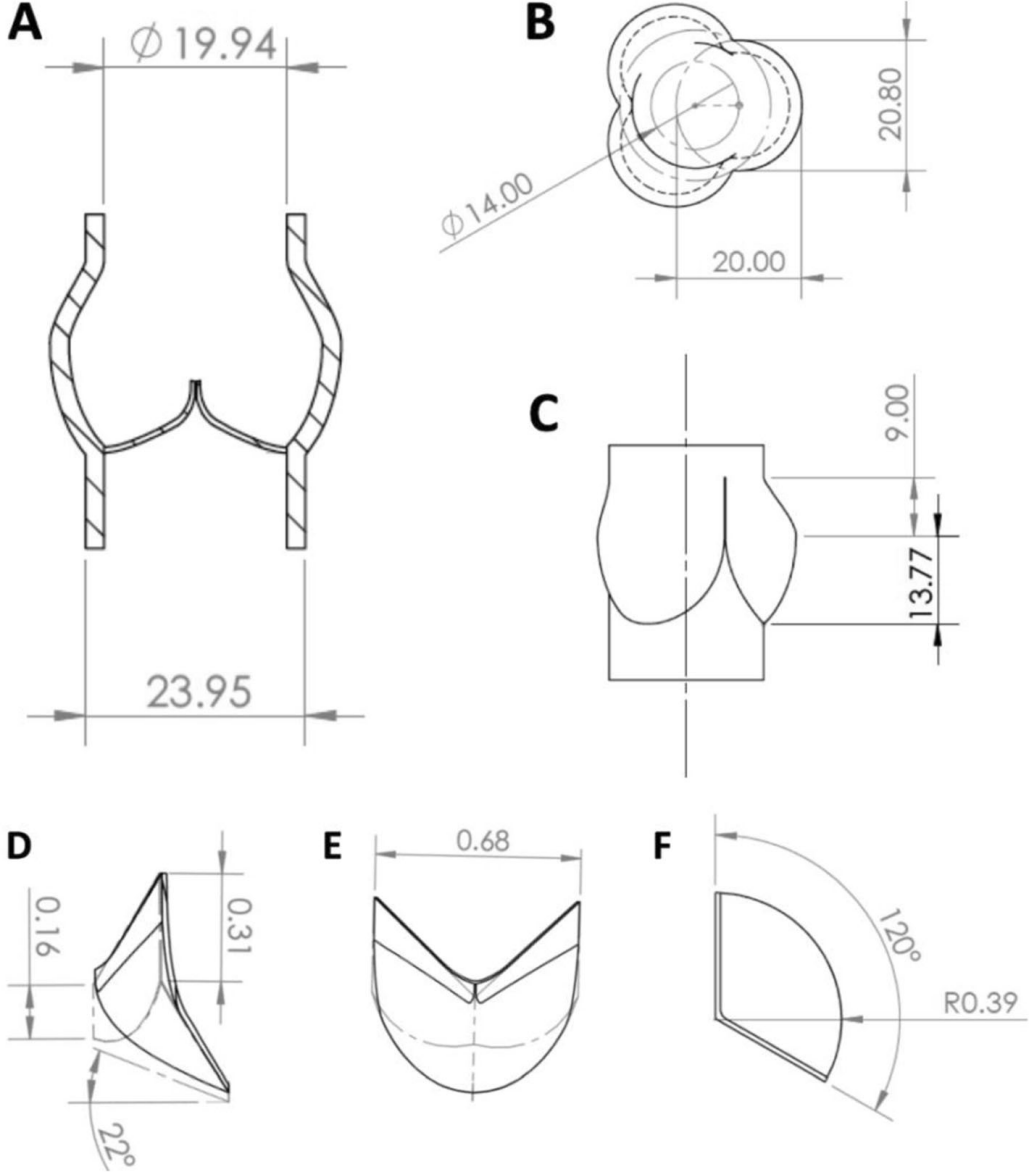
2D sketches of aortic root and valve leaflet geometry (mm). **a** Coronal view of the aortic root. Wall thickness 2 mm. **b** Cross-sectional View of aortic root. **c** Lateral view of aortic root. **d** Lateral view of valve leaflet. **e** Frontal view of valve leaflet. **f** Superior view of valve leaflet

**Fig. 3:**
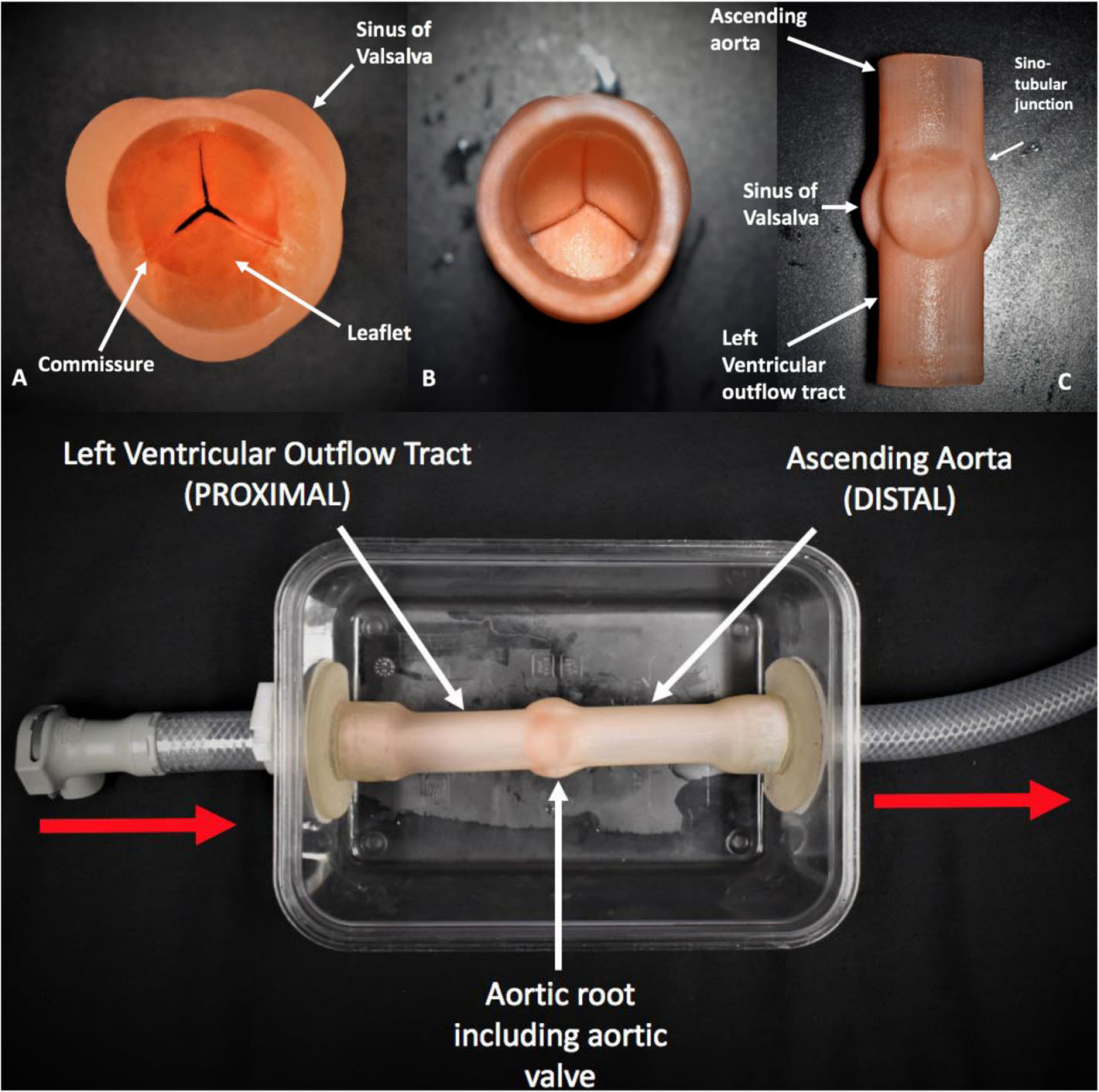
Top Panel: 3D printed tricuspid aortic valve phantom. **a** Superior view looking down the ascending aorta to visualize the aortic valve leaflets. **b** Inferior view looking up the left ventricular outflow tract to the base of the closed valve. **c** Lateral view of aortic valve phantom incorporating aortic root structures. Bottom Panel: Piping connected to phantom which attaches to the physiological MRI compatible pump. Red arrows show the direction of flow through the phantom

Previously published data using either pathology specimens or medical imaging were used to model the structure of the ‘normal’ tricuspid aortic valve with a CAD software package (SolidWorks, Waltham, Massachusetts, USA) (Figs. 1, 2) [1–7]. The design of the aortic root incorporated the sinuses of Valsalva, sinotubular junction, and ascending aorta. A cylinder with the dimension of the distal left ventricular outflow tract was modeled to complete the aortic valve phantom (Fig. 3).

**Fig. 4:**
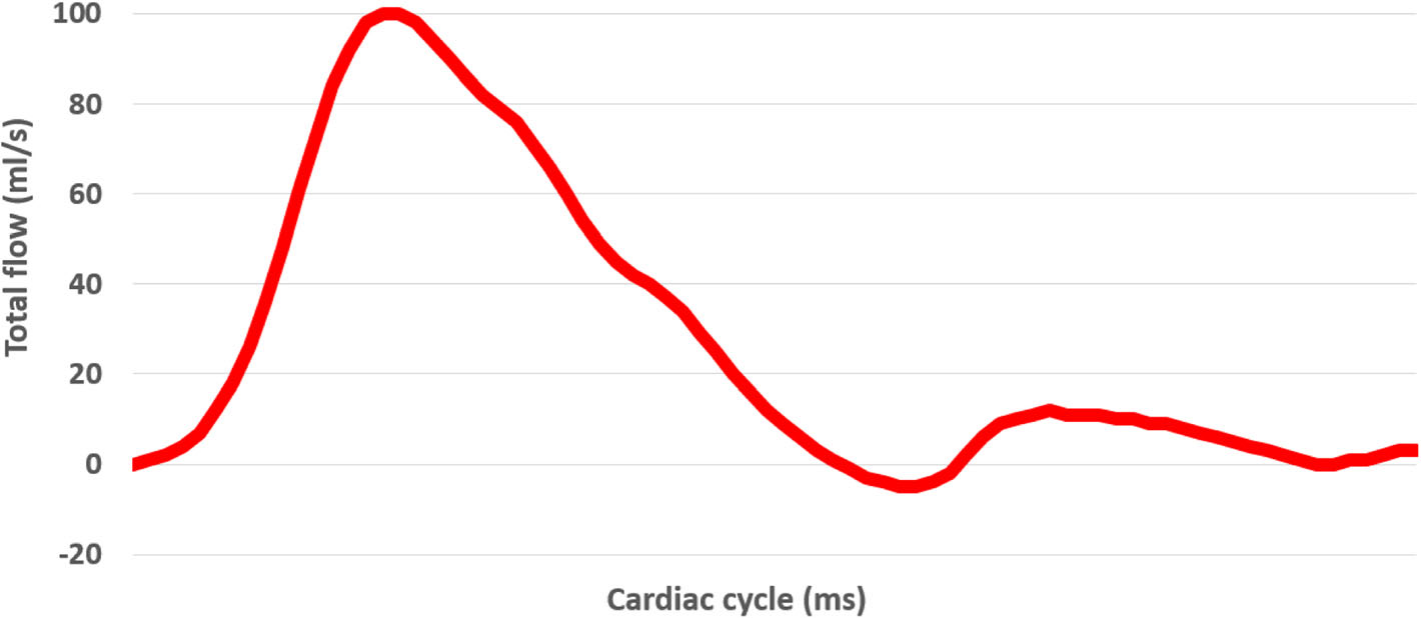
Normal aortic waveform programmed to flow pump over one cardiac cycle. Peak flow rate set at 100 mL/s

The stereolithography (STL) files were then generated and printed on a 3D printer (Objet 500 Connex 3, Stratsys Ltd., Eden Prairie, MN, USA). After multiple trials with various materials, a mixture of Agilus Clear and VeroWhite resin material was used to create the aortic walls and sinus and TangoPlus resin was used for the individual valve leaflets. The leaflet thickness was 0.6 mm. An MRI compatible flow pump (CardioFlow 5000, Shelley Medical Imaging Technologies, London, Ontario, Canada) with the capacity to mimic physiological flows was connected to the aortic flow phantom. The fluid reservoir, flow pump and phantom model were all connected in series on a single loop circuit. To mimic diastolic arterial pressure, the circuit between the phantom return line and reservoir was open and elevated such that the phantom sits 1088 mm below the water level. This setup results in a diastolic pressure equivalent to 80 mmHg at the phantom’s ascending aorta. The pump was programmed to generate a flow pattern, with a peak flow rate of 100 mL/s, simulating a normal aortic waveform (Fig. 4). The heart rate was set to 60 beats per minute. Prior to its introduction into the MRI scanner, the flow circuit was carefully tested on the bench, including graduated cylinder/stopwatch measurements to verify the accuracy of the pump. Following these preliminary tests, MRI data were acquired on a 3 T Prisma system (Siemens Healthineers, Erlangen, Germany).

MRI measurements included:

1. Cine imaging - to assess valve motion throughout the cardiac cycle
2. 2D phase contrast flow imaging – to measure flow velocity above and below the valve and assess for evidence of aortic stenosis (significant flow acceleration across the valve) or aortic regurgitation (retrograde flow) and to compare the data with ground truths.
3. 4D phase-contrast flow imaging – to visualize and analyze complex flow patterns using vector fields and path lines (Table 1).

**Table 1:** Pulse sequence parameters from the Digital Imagining and Communications in Medicine (DICOM) data for each of the modalities used

	Scan duration (minutes)	2D/ 3D	In-plane resolution (mm^2^)	Slice thickness (mm)	TR/TE (ms)	Field of View	Matrix size	Flip angle (°)	Venc (cm/s)
4D Flow	11.7 (in-plane GRAPPA acceleration factor = 3)	3D	1.6 × 1.6	1.6	48.6/3.5	200 × 156 × 64 mm^3^	128 × 102 × 40	7	100
Phase Contrast	0.5	2D	1.0 × 1.0	5 (single slice acquisition)	50.9/4.1	200 × 119 mm^2^	192 × 116	25	70
Cine (axial)	1.5	2D	1.0 × 1.2	3 (5 slices acquired)	58.8/3.3	200 × 119 mm^2^	192 × 99	12	n/a
Cine (coronal)	1.5	2D	1.2 × 1.3	3 (3 slices acquired)	57.5/3.3	224 × 133 mm^2^	192 × 99	12	n/a

## Results

**Fig. 5:**
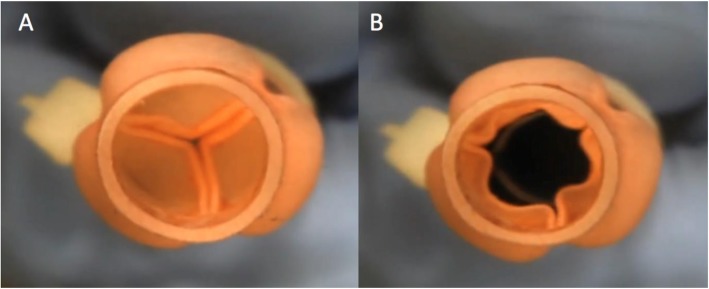
View from above the 3D printed aortic valve demonstrating the valve closing and opening in response to pulsatile flow. **a** Valve leaflets closing during diastole. **b** Valve leaflets opening during systole. (Corresponding video files attached – Additional file 1: Video S1)


A live video recording demonstrating the function of the 3D printed valve is provided in Fig. 5 (and Additional file 1: Video S1). Valve opening and closure during systole and diastole, respectively, is clearly visualized.

**Additional file 1: Video S1.** View from above the 3D printed aortic valve demonstrating the valve closing and opening in response to pulsatile flow.


**Fig. 6:**
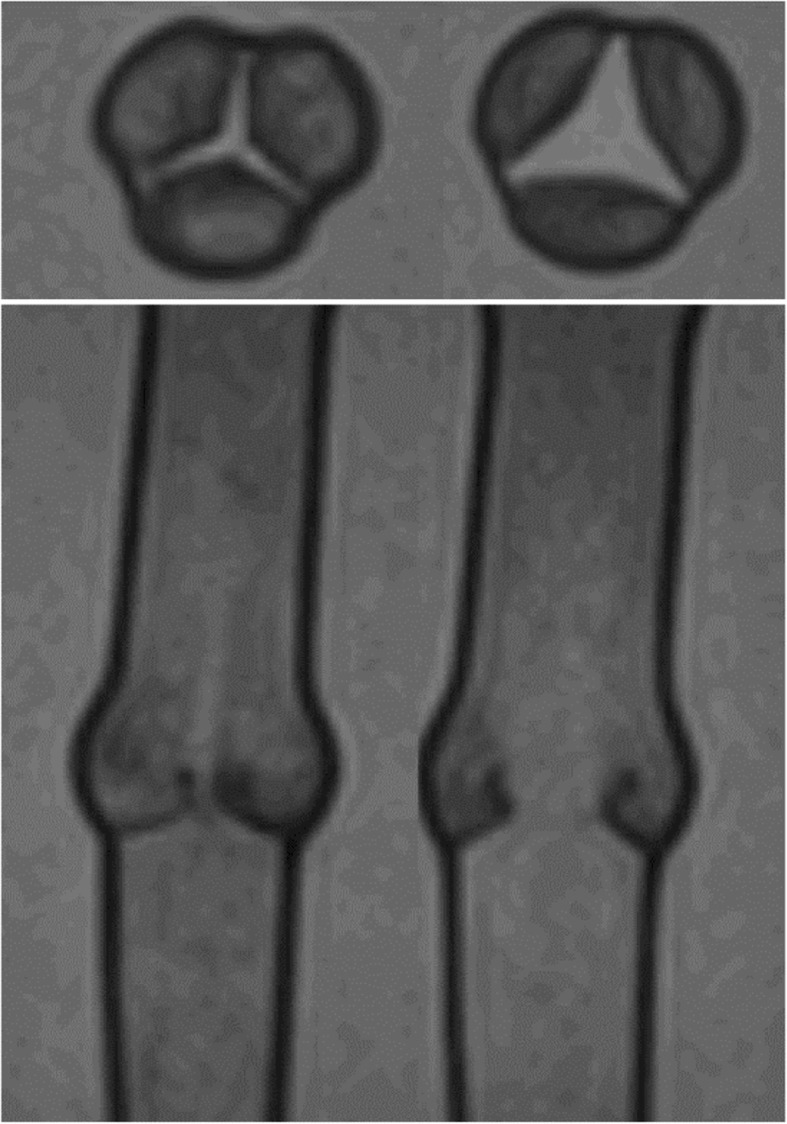
Cine MRI images showing aortic valve phantom in simulated systole in short and long axis planes. Left image shows the valve leaflets closed. Right image shows leaflets open. (Corresponding video files attached – Additional file 2: Video S2)

**Fig. 7:**
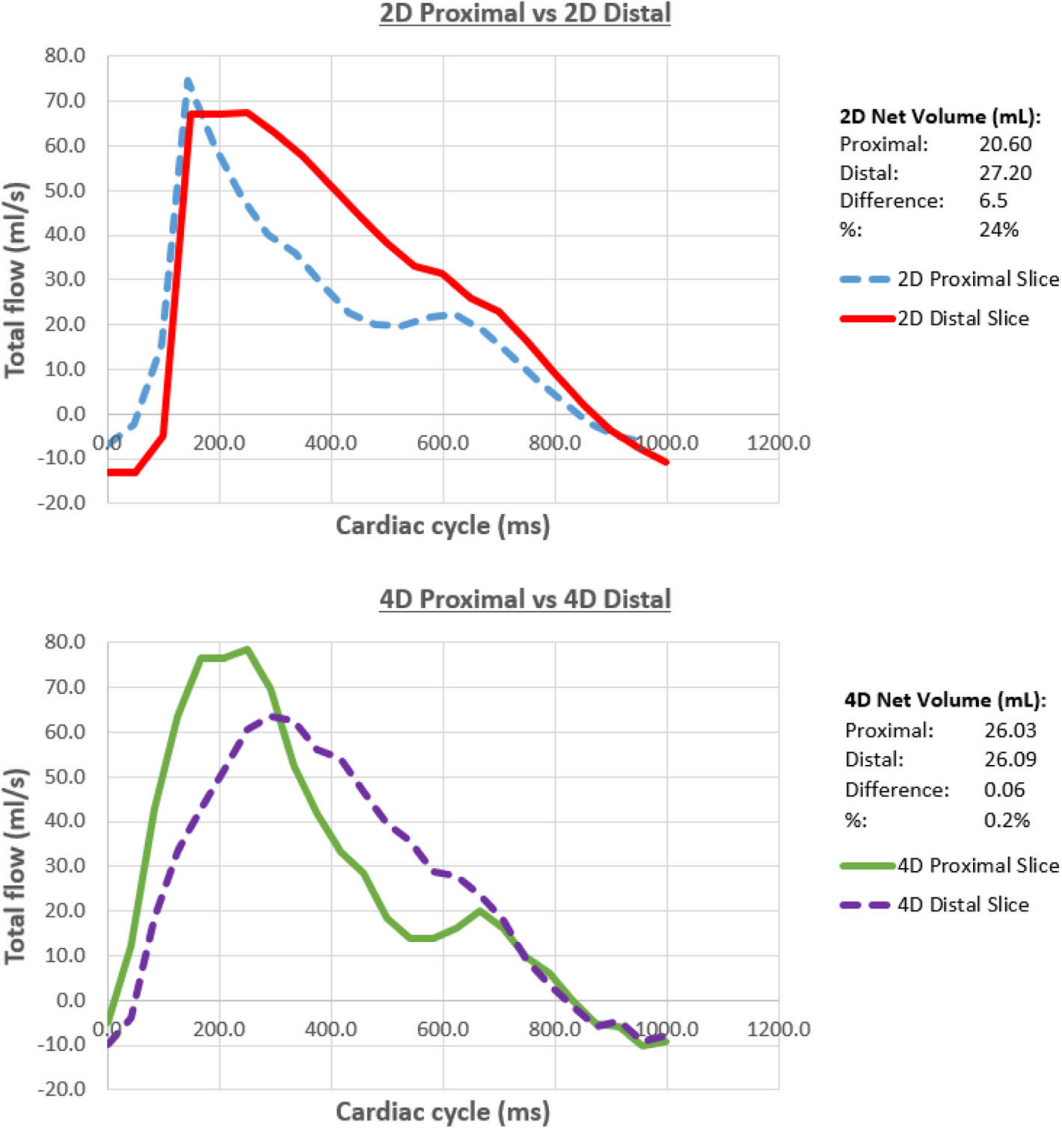
Correlation of blood flow volumes through the phantom proximal and distal to the aortic valve measured by 2D and 4D phase contrast imaging. There is better consistency of flow volumes in 4D than the 2D phase-contrast MRI measurements

Cine MRI data, providing both short and long axis views of the functioning valve are shown in Fig. 6 (and Additional file 2: Video S2). During systole the leaflets opened instantaneously allowing unobstructed flow through the valve and the leaflets closed effectively with cessation of systolic forward flow. Visualization and quantitative analysis of 4D Flow data was performed using a commercial software package (4D Flow, Siemens, Erlangen, Germany). Retrospective measurements of net flow through 2D planes placed proximal and distal to the valve were very consistent (26.03 mL/s and 26.09 mL/s, respectively). Flow waveforms measured at these two planes are provided in Fig. 7. Peak flow values proximal and distal to the valve were 78.4 mL/s and 63.3 mL/s, respectively. Measurements of net flow value agreed closely with the integral of the flow waveform provided to the CardioFlow 5000 (27.74 mL/s), confirming 4D flow acquisition in relation to the pump output.

**Fig. 8:**
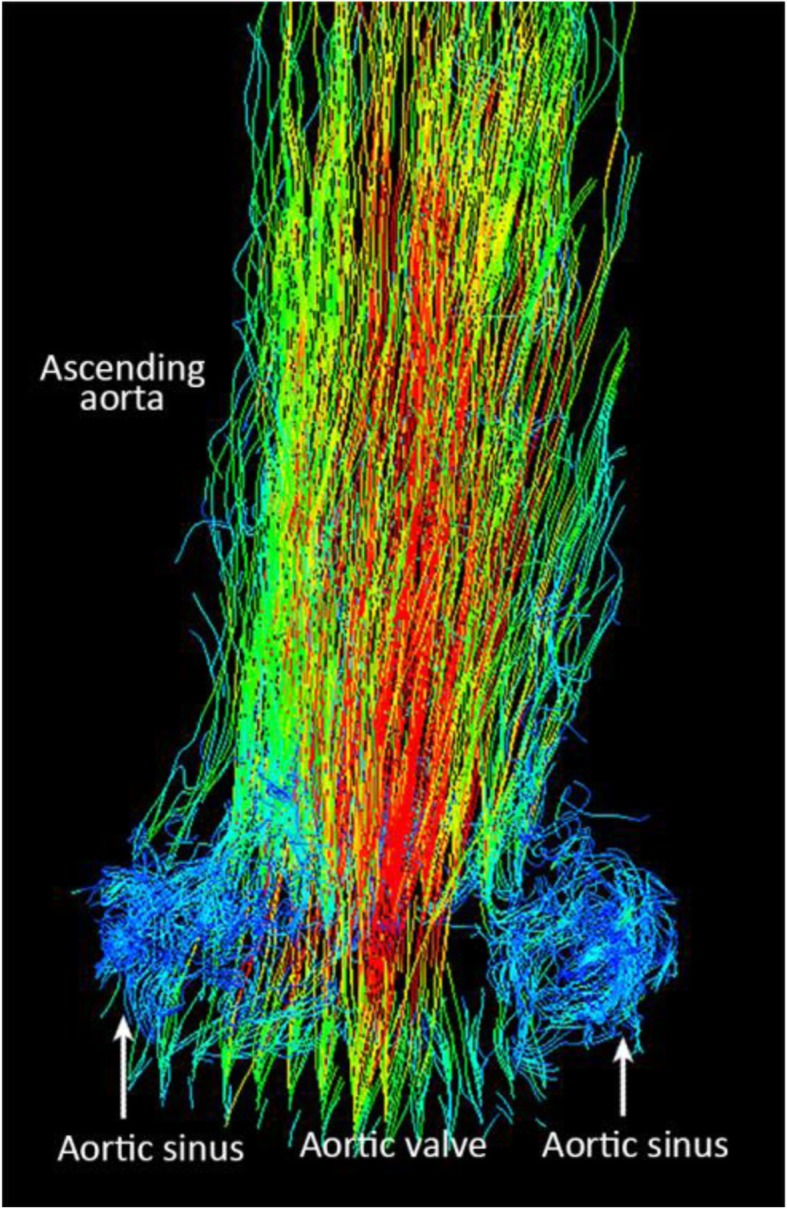
Particle tracing of flow from 4D phase-contrast MRI showing vortices in the aortic sinuses. Seeds were placed at the aortic valve orifice (Corresponding video file attached – Additional file 3: Video S3)

Particle traces of flow from 4D-phase contrast MRI data demonstrated flow through the valve into the ascending aorta, as well as vortices within the aortic sinuses, which are expected during ventricular diastole. There was a small degree of retrograde flow during diastole, suggestive of regurgitation. (Fig. 8 + Additional file 3: Video S3).

2D contrast imaging of flow volumes were less consistent than 4D with net flow proximal and distal to the valve measuring 20.60 mL and 27.20 mL, respectively. Peak flow values proximal and distal to the valve were 74.6 mL/s and 67.6 mL/s respectively.

## Discussion

The semilunar valves are uniquely structured to open and close without the aid of the chords and papillary muscles. The tricuspid semilunar valve with properly sized and shaped sinuses of Valsalva appears to be the ideal configuration for optimal hemodynamics, compared to mono or bicuspid variants [4, 8–10]. Any alteration in the number of valve leaflets, the size of the valve annulus and the size and shape of the sinuses may result in inadequate flow or turbulence across the valvular orifice and damage to the valve leaflets. Both aortic and pulmonary valve diseases are not uncommon and require replacement of the diseased valve with a prosthetic or bioprosthetic valve through surgery or intervention [11–13]..

3D printing of patient specific aortic root phantoms has previously been used successfully to develop procedural simulations for in vitro transcatheter aortic valve replacements (TAVR) and to quantitatively assess post-TAVR aortic root strain and potential incidence of paravalvular leak using computed tomography [14]. Our pilot study provides the first demonstration of 3D printing of flexible semilunar valves fabricated with CAD, followed by detailed assessments of function and flow using cine- and 4D Flow MRI.

The results generated further validate the efficacy of using additive manufacturing as a feasible and effective methodology to assess semilunar valve function. In addition, it has been shown that 4D flow MRI can be used in phantoms to investigate the complex flow patterns within the aortic root, which are crucial in evaluating valve leaflet closure and coronary artery perfusion. This method can support ongoing work in the field of computational -fluid dynamics (CFD), which has analyzed normal aortic valve function, in addition to simulating surgical repairs of the aortic root [15–18].

The workflow demonstrated in this paper can be applied to the investigation of: 1) the structural requirements for optimal function and durability of the semilunar valve and the sinuses of Valsalva, 2) the study of the hemodynamic changes in association with abnormal valve leaflet and sinus configuration such as various forms of bicuspid and quadricuspid valves, and 3) the effect of the dilated aortic root on aortic valve anatomy and function in systemic connective tissue diseases such as Marfan and Loeys-Dietz syndromes. Although the listed features can be assessed in living individuals, in-vivo studies are associated with numerous confounding factors such as ventricular function, heart rate, distal vascular resistance and anatomical variations. Inevitably this method requires a large study population to prove the given hypotheses. As any number of combinations of anatomical and hemodynamic variations can be fabricated with CAD and 3D printing, with the other confounding factors kept constant, the structural and functional importance of each component of the valve and sinus can be isolated and assessed in detail. Fabrication of individualized semilunar valve phantoms with computer and phantom flow dynamic studies may ultimately allow optimized design of bioprinted implantable valves. The experimental setting with phantoms can also be used for validation of the flow assessment tools including Doppler ultrasound, 2D- and 4D-flow MRI using precisely calibrated flow pumps. This methodology streamlines the process from prototyping to valve testing, reducing the time required to optimize the structure of the semilunar valve.

Potential areas for improvement of the demonstrated workflow include: quality of 3D print material, the CAD technique for valve design and the physiological fidelity of the flow pump and circuit. The current commercially available 3D print material used to generate the valve phantoms does not have identical elastic, strength or biochemical properties to human valve tissue and will remain a limitation of this method until improved materials are available. While we were able to fabricate the valve leaflets with 0.6 mm thickness with the current commercially available print material, the forthcoming materials allow for fabrication of valve leaflets with a much more realistic 0.3 mm thickness [9, 19]. The use of a compliance chamber within the circuit, instead of an open circuit, would also improve the ability to control diastolic pressures being a more accurate reflection of physiological conditions. Although MRI has been proven to be a useful and accurate tool for flow assessment, further validation of 2D- and 4D flow -MRI is required in a larger number of phantom studies. Echocardiography can also be used as an additional modality to confirm the finding found on MR. This would be particularly useful in in-vivo studies where MR availability may be limited.

### Future directions

Further experimentation with anatomically accurate phantoms will potentially advance our understanding of physiological valve function. With valve repairs being more desirable than replacements, particularly in the pediatric population; this methodology could potentially be used to assess the efficacy and durability of current novel aortic valve repair techniques prior to performing these complex procedures. Additionally, it may provide a safe platform to develop and validate future surgical valve repair techniques. Prior to patient translation, tissue engineering with in-vivo animal studies is the next step to validate this methodology. Tissue-engineered valves will address issues of biocompatibility and valve leaflet durability with the 3D-printed phantoms providing accurate geometric data for valve scaffolds with favourable flow dynamics.

## Conclusion

In this proof of concept study, we have demonstrated the ability to use existing geometric aortic valve data to generate physiological 3D-printed aortic valve phantoms and evaluate their function with cine- and 4D Flow MRI. This methodology could be used to improve our understanding of the function of the semilunar valves and develop the correct geometry to achieve optimal flow dynamics. It also supports quantitative assessment of specific factors affecting valve function, such as the number of valve leaflets, the configuration of the sinuses of Valsalva, dilatation of the aortic root or pulmonary trunk and coronary artery perfusion during leaflet coaptation. This technology can work synergistically with the promising tissue engineering research in the quest to develop optimal aortic valve replacements, which most closely reproduces the complex function of the normal aortic valve.

## Supplementary information

**Additional file 2: Video S2.** Cine MRI images showing aortic valve phantom in simulated systole in short (A) and long axis planes (B).

**Additional file 3: Video S3.** Particle tracing of flow from 4D phase-contrast MRI showing vortices in the aortic sinuses. Seeds were placed at the aortic valve orifice.

## Data Availability

All data generated or analysed during this study are included in this published article.

## Abbreviations

4D: 4-dimensional
CAD: Computer-aided design
CFD: Computational fluid dynamics
MRI: Magnetic resonance imaging
STL: Stereolithography
TAVR: Transcatheter aortic valve replacement
